# Supplementary material for: Cholinergic-estrogen interaction is associated with the effect of education on attenuating cognitive sex differences in a Thai healthy population
Source: PLoS One. 2023 Jul 20;18(7):e0278080. doi: 10.1371/journal.pone.0278080 (PMC10358962; doi:10.1371/journal.pone.0278080)
Supplement: Attachment — Submitted filename: PONE D 2230636 comments.docx [file pone.0278080.s003.docx]

This manuscript hypothesizes that education decreases the cognitive differences between genders. To examine this an experimental design was developed in humans, especially those who studied above primary level of education and those who didn’t. The authors use of Bayes factor as a statistic is impressive and the results that span behavioural, biological and bioinformatical domains showcases the strong experimental design. has

1. These findings could be discussed in light of following papers, as some studies have found marginal improvements with education and gender based changes in cognitive performances. - [**https://www.ncbi.nlm.nih.gov/pmc/articles/PMC4608774/**](https://www.ncbi.nlm.nih.gov/pmc/articles/PMC4608774/) ; Efstathios D. Gennatas, Brian B. Avants, Daniel H. Wolf, Theodore D. Satterthwaite, Kosha Ruparel, Rastko Ciric, Hakon Hakonarson, Raquel E. Gur, Ruben C. Gur. **Age-Related Effects and Sex Differences in Gray Matter Density, Volume, Mass, and Cortical Thickness from Childhood to Young Adulthood**. The Journal of Neuroscience, 2017; 37 (20): 5065 DOI: [10.1523/JNEUROSCI.3550-16.2017](http://dx.doi.org/10.1523/JNEUROSCI.3550-16.2017)
2. Lines 63-65 are not clear, What is the conclusion of that study?
3. Also, a population with age range of 22-70 was used here, this is a big range for age. As some are young adults and some are aging.
4. WISC is used in set shifting, capability of a person to change his attention based on new rules, it is not a memory test, Although it showcases frontal lobe function, especially the DLPFC, still many other forms of cognitive/memory testing could be done and would have elevated the paper. An explanation to this in the discussion would help, please cite other papers where WCST was used alone and the reliability of WCST for cognitive measurements as a stand-alone test.
5. Line 176-177, the age differences between groups, meaning the tertiary and secondary educated groups may just perform well because they are significantly lower in age compared to primary educated group?
6. The graphs are not consistent, In one graph, males are followed by females, while in others, it’s the reverse.
7. Also, did the authors make a comparison of females in tertiary education to primary education? Was there a difference? Careful examination of data with multiple comparisons are needed in primary vs. secondary educated females, males across all three parameters shown.
8. Also, a discussion on why the other two parameters of WCST were not showing results needs to be discussed? Please refer to any previous publications that showed such discrepancies if any? If not, please consider adding more numbers to the study.
