# Supplementary material for: Cholinergic-estrogen interaction is associated with the effect of education on attenuating cognitive sex differences in a Thai healthy population
Source: PLoS One. 2023 Jul 20;18(7):e0278080. doi: 10.1371/journal.pone.0278080 (PMC10358962; doi:10.1371/journal.pone.0278080)
Supplement: Attachment — Submitted filename: Response to Reviewers .docx [file pone.0278080.s004.docx]

5. Review Comments to the Author

**Reviewer #1**: This study aims at examining the effects of education level on cognitive sex differences. The authors classify participants at different ages to three categories based on their education levels and then examine the differences in various WCST scores between males and females. They have also examined the sex-specific neurotransmission-related protein expression profiles and tried to link it to the sex-related differences in behavioural measurements. Understanding the differences in cognitive abilities between males and females and delineating the effects of environmental factors are important and therefore the main goal of this study has merit. However, there are various uncontrolled factors in this study, which make it inconclusive. The analytical approach also hinders reaching a clear conclusion.
Major points:

1- In this study, the education level (primary, secondary,…) has been considered as an isolated factor, however various other factors (such as socioeconomic status) might be strongly associated with the education level. The authors also cite previous studies indicating that socioeconomic status might significantly affect cognitive development and performance in cognitive tasks. This issue has not been controlled in this study.

**Author Response:**

When comparing sex differences in WCST sub-scores at each educational level, age as a confounder was controlled. The model diagnostic was then performed to ensure that the model fits the data. Furthermore, the Bayes factor (BF) was employed as direct evidence to support the alternative hypothesis [ref 38]. This is done to ensure the validity of our findings. However, because other confounders such as socioeconomic status and profession were not controlled for, this study may have limitations, which have been noted in the revised manuscript.

2- The analytical approaches should be accompanied by direct comparison of the groups within the same statistical analyses (multi-factorial ANOVA). While assessing the evidence for a particular hypothesis for each separate group is informative, direct comparison of groups appears necessary. In other words, the effects of Age, Sex and Education factors should be examined with analytical approaches (same statistical analysis) to confirm the significance (or absence of significance) for the main effects and their interactions.

**Author response:**

The results of multi-factorial ANOVA revealed that sex, age and education level (P≤0.05) had significant main effects on two of the five WCST-sub scores %Corrects and %Errors. Aside from that, there were significant interaction effects of sex by age (P＜0.01) on scores %Corrects, %Errors and Category Completed, as well as significant age by education interaction effect on 1^st^ Category (P＜0.05). This information has been added to the revised manuscript (lines 195-201).

3- The examination of differentially expressed proteins between males and females, is informative, however it is simply a correlational analysis and does not establish any causal interaction between the cognitive sex difference and alterations in these proteins. Elsewhere in the manuscript a causative association is claimed “we discovered that cholinergic signalling may be at the root of the effect of education on attenuating cognitive sex differences” or “Cholinergic estrogen interaction underpins the effect of education on attenuating cognitive sex differences in a Thai healthy population”. However, the experimental approaches and results do not establish a causative link and conclusions are not supported.

**Author response:**

We agree that it is inappropriate to imply causality and have changed the manuscript throughout to avoid that suggestion.

Minor issues
1- Although they have used a computerized version of the WCST, the participants’ response time (RT) has not been measured/reported. RT could have been a more sensitive measure to show the sex-related differences (Feizpour et al. Psychology of Music 2018).

**Author response:**

We appreciate the reviewer’s suggestion and agree this is a limitation which we have acknowledged in the discussion (lines 378-382). This will be used as a measure in further study.

2- Literature review in the Introduction and Discussion needs to cite and discuss relevant studies in the context of the WCST.

**Author response:**

The Introduction and Discussion of the manuscript have been revised, citing and discussing relevant WCST studies.

3- Figure 1 can be improved in terms of clarity and quality. Currently, the data points cannot be easily detected.

**Author response:**

The clarity and quality of the original figure should be no problem, with the data points can be easily seen. However, we have improved its clarity and updated it in the revised manuscript.

**Reviewer #2:** This manuscript hypothesizes that education decreases the cognitive differences between genders. To examine this an experimental design was developed in humans, especially those who studied above primary level of education and those who didn’t. The authors use of Bayes factor as a statistic is impressive and the results that span behavioural, biological and bioinformatical domains showcases the strong experimental design. Has

1. These findings could be discussed in light of following papers, as some studies have found marginal improvements with education and gender based changes in cognitive performances. - https://www.ncbi.nlm.nih.gov/pmc/articles/PMC4608774/ ; Efstathios D. Gennatas, Brian B. Avants, Daniel H. Wolf, Theodore D. Satterthwaite, Kosha Ruparel, Rastko Ciric, Hakon Hakonarson, Raquel E. Gur, Ruben C. Gur. Age-Related Effects and Sex Differences in Gray Matter Density, Volume, Mass, and Cortical Thickness from Childhood to Young Adulthood. The Journal of Neuroscience, 2017; 37 (20): 5065 DOI: 10.1523/JNEUROSCI.3550-16.2017

**Author response:**

We appreciate the reviewer’s suggestion, and these two papers have been studied and cited in the revised manuscript as ref.51 and ref.61.

2. Lines 63-65 are not clear, What is the conclusion of that study?

**Author response:**

According to Bloomberg and others [ref 18], males in UK had higher fluency scores only in the low education group (less than high school diploma). There was evidence of greater fluency scores in females in the high education group (high school diploma and higher), particularly in those born later. This finding implied that schooling, as well as secular changes in education level across birth cohorts, had a role in determining cognitive performance in females. We have now included a clarification to this effect in the text (lines 71-76).

3. Also, a population with age range of 22-70 was used here, this is a big range for age. As some are young adults and some are aging.

**Author response:**

In an attempt to obtain a sample that is approximately representative of the population, the sample covers a range of subjects with varied ages, which could have increased the variability of the sample. To ensure the validity of the study, age was controlled in the process of the data analysis. This limitation has also been mentioned in the discussion of the revised manuscript (lines 369-374).

4. WISC is used in set shifting, capability of a person to change his attention based on new rules, it is not a memory test, Although it showcases frontal lobe function, especially the DLPFC, still many other forms of cognitive/memory testing could be done and would have elevated the paper. An explanation to this in the discussion would help, please cite other papers where WCST was used alone and the reliability of WCST for cognitive measurements as a stand-alone test.

**Author response:**

The standardization, validity and reliability of WCST as a stand-alone cognitive assessment in healthy adults of various ages and education levels have been established [ref 66]. However, a battery of cognitive tests is required to unveil the effects of education on different domains of cognition. We have discussed this in the revised manuscript (lines 317-320).

5. Line 176-177, the age differences between groups, meaning the tertiary and secondary educated groups may just perform well because they are significantly lower in age compared to primary educated group?

**Author response:**

When comparing the WCST scores of subjects from the two educational groups. Females with a higher education level outperformed those in the primary education group in only two WCST sub-scores: %Corrects and %Errors. While there is limited evidence to show differences in WCST sub-scores in males across the two educational levels. Age as a covariate was controlled in the calculation.

6. The graphs are not consistent, In one graph, males are followed by females, while in others, it’s the reverse.

**Author response:**

The graph in Figure 1 of this manuscript was created using the default setting of R package “flexplot”. We agree with the reviewer’s suggestion. So, the graphs have been revised.

7. Also, did the authors make a comparison of females in tertiary education to primary education? Was there a difference? Careful examination of data with multiple comparisons are needed in primary vs. secondary educated females, males across all three parameters shown.

**Author response:**

Yes, we did. Females with secondary and tertiary education performed significantly better in scores %Corrects and %Errors (BF＞100) than those in the lower education group, and moderate evidence for such difference in the score 1^st^ Category (BF=4.55). While limited evidence indicated differences in scores of %Corrects (BF=4.79), %Errors (BF=4.43), and 1^st^ Category (BF=3.48) in males across the two education levels. This has been added to the revised manuscript (lines 227-232).

8. Also, a discussion on why the other two parameters of WCST were not showing results needs to be discussed? Please refer to any previous publications that showed such discrepancies if any? If not, please consider adding more numbers to the study.

**Author response:**

The other two WCST scores, Category Completed and PE, did not demonstrate significant sex differences in the current research, which contradicts earlier studies [ref 16, 65]. Perhaps the relative low number of subjects following stratification by education in each education group prevent us from detecting sex differences in these two scores [ref 16] (lines 308-312). The limitation of the moderate sample size has been noted in the text (lines 387-388).
