# Supplementary material for: Cholinergic-estrogen interaction is associated with the effect of education on attenuating cognitive sex differences in a Thai healthy population
Source: PLoS One. 2023 Jul 20;18(7):e0278080. doi: 10.1371/journal.pone.0278080 (PMC10358962; doi:10.1371/journal.pone.0278080)
Supplement: Attachment — Submitted filename: Response to reviewers for 2nd revision.docx [file pone.0278080.s005.docx]

6. Review Comments to the Author

**Reviewer #3**: The article titled "Sex Differences in Cognitive Performance: Potential Compensatory Mechanisms Revealed by Proteomic Analysis" presents a study investigating sex differences in cognitive performance and potential compensatory mechanisms using proteomic analysis. The article is well-structured, providing detailed information on the study's background, methods, results, and discussion.
The study involved 135 subjects, 70 males, and 65 females, with a mean age of 57.75±10.35 years. The authors explored the effects of education on sex differences in cognitive performance by administering the Wisconsin Card Sorting Test (WCST) and comparing the results between males and females in different education groups. They found that education had potential compensatory effects on three out of the five WCST sub-scores: %Corrects, %Errors, and 1st Category. In the primary education group, males performed better in %Corrects and had lower %Errors, while females completed the first category in fewer trials. However, in the higher education group, sex differences in those scores reversed, with females performing better in %Corrects and males completing the first category in fewer trials. The authors suggest that education may play a role in compensating for sex differences in cognitive performance, although further research is needed to confirm this hypothesis.
In addition to the behavioral tests, the authors used a label-free quantitative proteomics approach to identify differentially expressed proteins (DEPs) between males and females. They found that 11 DEPs were present in both education groups, with all but one upregulated in females. The authors then investigated the protein-protein interactions of these DEPs and their relationship with cognitive function. They found that LRP4, TTR, TRIM38, and PP2B directly interacted with nAChR3, a subunit of nicotinic acetylcholine receptor that positively regulates cognition. On the other hand, CD34, SERPING1, HSD17B1, KNG1, and TTR were indirectly connected to the cholinergic pathways through interaction with estrogen, suggesting that the cholinergic-estrogen interaction may have an influence on cognitive processes.
The significance of this study lies in its exploration of the potential effects of education on sex differences in cognitive performance and their relationship with differentially expressed proteins between men and women. The study provides insights into the complex interactions between education, sex, and cognitive function, and sheds light on potential molecular mechanisms underlying sex differences in cognitive performance.
The findings of the study suggest that education may have compensatory effects on sex differences in cognitive performance, particularly in the WCST sub-scores of %Corrects, %Errors, and 1st Category. The study also identified 11 differentially expressed proteins between men and women, most of which were upregulated in females, and highlighted the potential roles of cholinergic-estrogen interactions in cognitive processes.
These results have important implications for understanding sex differences in cognitive function and developing interventions to improve cognitive performance in both sexes. By identifying the molecular mechanisms underlying these differences, future research may be able to develop targeted interventions that leverage the compensatory effects of education and modulate the cholinergic-estrogen pathway to improve cognitive function in both men and women.

Overall, the article provides valuable insights into the potential compensatory mechanisms underlying sex differences in cognitive performance. The use of proteomics analysis adds a novel dimension to the study, providing a more detailed understanding of the biological mechanisms underlying cognitive performance. However, the study has some limitations, including the relatively small sample size, the cross-sectional design, and the lack of information on other potential confounding factors such as lifestyle factors or medical conditions. Nevertheless, the study provides a valuable contribution to the literature on sex differences in cognitive performance and sets the stage for further research in this area.

Major concern
1. Sample size: The sample size of the study is relatively small, which limits the generalizability of the findings. Larger and more diverse samples would provide a more robust assessment of the relationships among cognitive performance, sex, education, and protein expression.

**Author response:** We discussed the limitations of this study in the last paragraph of the Discussion section, which included the moderate sample size (line 409), and the proportion of middle-aged and elderly subjects was much higher than that of young adults, with the young participants clustering in the higher education group (lines 389-391). We agree that a larger and more diverse sample size would provide more robust results, and had revised our manuscript to show that our findings provided a preliminary indication of potential compensatory effects of education underlying cognitive sex difference attenuation (lines 41-43), and that further generalizing the results of our study should be done with caution (lines 409-410).

2. Methodological limitations: The study used a cross-sectional design, which makes it challenging to draw causal inferences about the relationships among the variables under investigation. Longitudinal studies would be useful for tracking changes in cognitive performance and protein expression over time and for evaluating potential causal relationships.
 **Author response:** We appreciate the reviewer’s suggestion and agree this is a limitation which we have acknowledged in the discussion (lines 405-409). This will be used as the methodology in further study.

3. Statistical analysis: Although the study used Bayesian statistical methods, the presentation of the results could be improved by providing more detailed information on the Bayesian analyses, such as prior distributions and sensitivity analyses.

**Author response:** The default prior used in the general linear model is the Jeffreys-Zellner-Siow (JZS) priors [ref 40], with a default r value of 0.353 applied. After sensitivity analyses [ref 39], the results remain robust. This information has been added to the revised manuscript (lines 208-209, 221-222).

Minor concerns
1. Clearer headings and subheadings: The headings and subheadings in this article are a bit confusing and could be made clearer. For example, the section on demographic data could be titled "Demographic Characteristics of the Study Population" to better convey what the section is about.

**Author response:** We appreciate the reviewer’s suggestion and changed this subheading accordingly (line 184).

2. Improved organization: The article would benefit from a clearer organization of ideas. For instance, the section on the effects of education on sex differences in cognitive performance could be restructured to present the findings in a more coherent manner. The section could start with an introduction explaining why the effects of education on cognitive performance were investigated, followed by a clear description of the results and a discussion of the findings.

**Author response:** The rationale of why the effects of education on cognitive performance has been presented on the Introduction section (lines 70-77); the sub-section “Effects of education on sex differences in cognitive performance” in the Results section described the findings from our data analysis (line 208-236), and the related discussion was made in the Discussion section (lines 266-331).

3. Improved clarity: Some of the language used in the article is a bit difficult to understand. For example, the phrase "age differences between males and females in primary, secondary and tertiary education groups were supported by weak and substantial evidence, respectively" is confusing. A clearer way to say this might be "The study found that there were significant age differences between males and females in the secondary and tertiary education groups, but not in the primary education group."

**Author response:** We appreciate the reviewer’s suggestion and revised this unclear sentence accordingly (lines 187-189).

4. More detailed discussion: The discussion section of the article is quite short and could benefit from more detailed analysis of the findings. For example, the article could explore why females in the higher education group performed better on certain cognitive tasks than males, whereas males in the lower education group performed better.

**Author response:** In the Discussion section, why females in the higher education group performed better than males on certain cognitive tasks than males has been discussed (lines 287-299, 315-319). Regarding to why males in the lower education group performed better, the total errors of WCST reflect non-specific cognitive impairments, and another study we conducted suggested that females might be more susceptible to the excitotoxicity induced by WCS [ref 59]. This, combined with the loss of estrogen’s neuroprotection effects after menopause [ref 60, 61, 62], led to loss of brain environment homeostasis and impaired cognitive function in elderly female subjects [ref 59], as indicated by their cognitive performance (more errors). This has been added in the Discussion section (lines 299-306).

5. Address limitations: The limitations of the study should be addressed in the discussion section, and the authors should acknowledge any potential sources of bias or error in their study.

**Author response:** We have stated the limitations of this research in the last paragraph of the Discussion section and acknowledged potential sources of bias or error in the study (lines 387-410).

6. Clearer conclusions: The article's conclusions could be made clearer and more concise. The article could end with a summary of the main findings and their implications, along with suggestions for future research.

**Author response**: We appreciate the reviewer’s suggestion and improved the conclusion of this study (lines 413-415, 419-422).

7. Consider alternative explanations: The authors should consider alternative explanations for their findings, and discuss other factors that may influence cognitive function, such as lifestyle factors, socio-economic status, and cultural differences.

**Author response**: We appreciate the reviewer’s suggestion. Aside from education, other environmental factors that have been linked to cognitive sex differences include lifestyle factors [ref 73]，socio-economic status (SES) [ref 74, 75], and cultural differences [ref 76, 77]. Of these, SES has been proposed to be a factor in the discrepancy of sex difference in cognitive aging [ref 75]. However, such factors were not investigated in the current study. To further understand the connections between environmental factors and cognitive sex differences, additional research considering more factors related to the sex discrepancy in cognitive function is required. This has been added in the revised manuscript (lines 331-338).

Overall, the article contains valuable findings, but could benefit from some improvements in organization, clarity, and depth of analysis.
